# Construction and application of medication reminder system: intelligent generation of universal medication schedule

**DOI:** 10.1186/s13040-024-00376-y

**Published:** 2024-07-15

**Authors:** Hangxing Huang, Lu Zhang, Yongyu Yang, Ling Huang, Xikui Lu, Jingyang Li, Huimin Yu, Shuqiao Cheng, Jian Xiao

**Affiliations:** 1 Department of Pharmacy, Xiangya Hospital, Central South University, NO.87, Xiangya Road, Changsha, 410008 Hunan Province China; 2 Institute for Rational and Safe Medication Practices, National Clinical Research Center for Geriatric Disorders, Xiangya Hospital, Central South University, Changsha, 410008 Hunan China; 3 College of Medicine, Wuhan University of Science and Technology, Wuhan, 430000 Hubei China; 4 Department of Pharmacy, The Second People’s Hospital of Beihai, Beihai, 536000 Guangxi China

**Keywords:** Universal medication schedule, Medication reminder system, Chronic disease service, Minimum interval between two drugs in a day, Medication guide

## Abstract

**Background:**

Patients with chronic conditions need multiple medications daily to manage their condition. However, most patients have poor compliance, which affects the effectiveness of treatment. To address these challenges, we establish a medication reminder system for the intelligent generation of universal medication schedule (UMS) to remind patients with chronic diseases to take medication accurately and to improve safety of home medication.

**Methods:**

To design medication time constraint with one drug (MTCOD) for each drug and medication time constraint with multi-drug (MTCMD) for each two drugs in order to better regulate the interval and time of patients’ medication. Establishment of a medication reminder system consisting of a cloud database of drug information, an operator terminal for medical staff and a patient terminal.

**Results:**

The cloud database has a total of 153,916 pharmaceutical products, 496,708 drug interaction data, and 153,390 pharmaceutical product-ingredient pairs. The MTCOD data was 153,916, and the MTCMD data was 8,552,712. An intelligent UMS medication reminder system was constructed. The system can read the prescription information of patients and provide personalized medication guidance with medication timeline for chronic patients. At the same time, patients can query medication information and get remote pharmacy guidance in real time.

**Conclusions:**

Overall, the medication reminder system provides intelligent medication reminders, automatic drug interaction identification, and monitoring system, which is helpful to monitor the entire process of treatment in patients with chronic diseases.

**Supplementary Information:**

The online version contains supplementary material available at 10.1186/s13040-024-00376-y.

## Introduction

Chronic diseases and illnesses are increasing globally, and they have become a major public health burden on the economic and social development of the country. Many patients with chronic conditions, especially those with multiple coexisting conditions, may require multiple medications daily to manage their condition [1], and strict adherence to medication regimens is crucial for optimal outcomes [2]. However, a study has reported that medication noncompliance is common in patients with chronic diseases; nearly 75% of patients with medication noncompliance do not achieve optimal blood pressure [3]. This adversely affects the therapeutic outcomes, causes different degrees of major consequences and also increases the risk of complications [4].

The decline in compliance often manifests itself in behaviors such as not taking medication at the prescribed time, not taking medication, or interrupting medication [5], which has serious adverse effects on chronic diseases, especially hypertension and diabetes, whose treatment regimens are closely associated with chronopharmacology. Improved adherence may have a greater impact on the health of patients with chronic diseases than any new therapy discovery [4]. In 2008, the Institute of Medicine (IOM) introduced the concept of the universal medication schedule (UMS) in a report that describes when to take medications by assigning four clear times (morning, noon, evening, and bedtime) [6]. This single, standardized way of describing medications can help patients more clearly understand, organize, and simplify their medication regimens and improve medication adherence to the solid dosage forms they use daily. Multiple trials have demonstrated that UMS improves the understanding of the medication regimens of the patient, thereby facilitating their proper use of medications [7].

Although there is great potential for UMS in solid formulations [8], it is yet to be optimized for daily use in outpatient clinics and for patients with different lifestyles. Present methods used to improve medication adherence are rapidly evolving with the help of emerging technologies, such as apps and smart devices, as an adjunct to patient medication management, in addition to traditional outpatient medication education [9]. However, most apps and smart devices focus their functions on medication education, providing patients with medication information and medication alerts [10, 11], improving medication adherence through instant communication [12], and real-time monitoring [13, 14]. They improve patient medication adherence but are unable to provide personalized medication plans for patients. Charles Wachira et al. designed and developed a guideline encoding and execution engine using JSON format and an algorithm for dispensing schedule optimization to obtain optimized medication schedules for guideline-recommended medication regimens via computer-aided output [15]. However, the design was not examined for drug interactions on prescriptions and lacks evaluation of real data from the clinical setting, and further refinement is still required. Therefore, developing technologies that can assist outpatient clinics to better personalize UMS for patients is crucial.

The purpose of this study is to construct a medication reminder system for the intelligent generation of UMS. Firstly, the database of rational drug use is constructed by obtaining information from drug instructions, pharmacopoeia, evidence-based medicine tools, and combining with chronopharmacology and so on. Secondly, build a medical staff operator that can query the drug information in the database after the patient’s prescription entering and combine it with the patient’s daily life (such as sleep time and meal time) to intelligently allocate the time and order of multiple drugs. Finally, the patient terminal that can accurately remind patients to take medication according to the allocated time for taking medication is constructed, so as to improve patients’ compliance with medication, ensure the safety, effectiveness, economy and rationality of patients’ medication, and reduce the occurrence of adverse drug reactions and drug-induced diseases.

## Methods

### Data collection and quality control

The catalog of pharmaceutical products (approved drugs) and its instructions was obtained from the National Medical Products Administration of China (https://www.nmpa.gov.cn/datasearch/home-index.html#category=yp) (as of December 7, 2021). Pharmaceutical product is defined as ingredients, specifications, or manufacturers when there is an inconsistency between any of them. Pharmaceutical product instructions contain various fields and hence was difficult for both medical professionals and consumers to access for accurate and quick access. The drug interaction data were obtained from drugs.com in addition to the pharmaceutical product instructions (Drugs.com, Drug Interactions Checker, 2021, https://www.drugs.com/drug_interactions.html, Accessed 30 April 2021). Subsequently, we developed a text extraction tool for extracting information from drug manuals and texts related to drug interactions. This tool also incorporates natural language processing capabilities and is capable of handling text content in both Chinese and English. The extracted information was stored in a Microsoft Excel sheet for further processing and management. Finally, we imported the processed information into a web database to ensure that users can easily access and retrieve this information.

### Database content and usage

#### Basic drug information

**Fig. 1:**
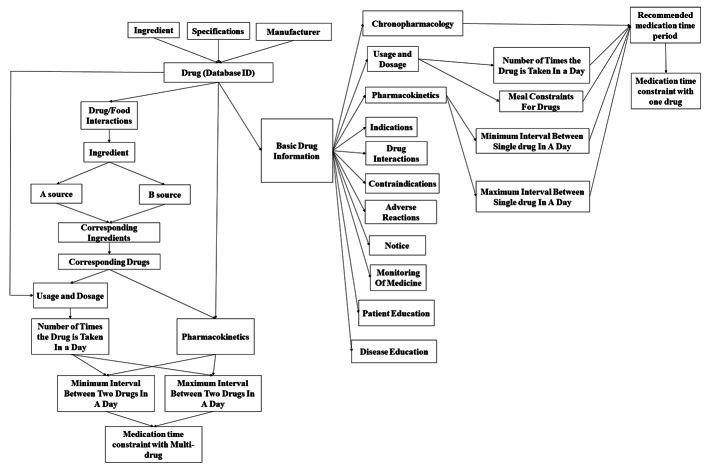
Interaction of main ontology classes in medication reminder system. A source, from the Drugs.com. B source, from pharmaceutical product instructions. Database ID, each pharmaceutical product has a database identity card in the database

All pharmaceutical products were marketed drugs approved by the National Medical Products Administration of China. The usage, dosage, pharmacokinetics, indication, drug interactions, and adverse reaction were derived from pharmaceutical product instruction, monitoring of medicine, chronopharmacology, patient education, and disease education were derived from original research that retrieved in PubMed. As shown in Fig. 1, these contents or information constituted basic drug information. The specifics of basic drug information are listed in Supplementary material S1.

#### Data of medication time constraint with one drug (MTCOD)

In clinical practice, patients usually decide the time to take medication based only on the number of times it has to be taken. However, this can be easily misunderstood by patients because taking a dose every 8 h and 3 times a day can have different time intervals between them [16]. Moreover, the timing of medications is also related to meals. For example, metformin taken by individuals with diabetes should be taken along with a meal. chronopharmacology, such as in the case of hypertension, is characterized by “two peaks and one valley,” i.e., two peaks from 9 am to 11 am and 4 pm to 6 pm, and one valley from 2 am to 3 am [17].

If a drug has to be taken many times in a day, a minimum interval should be present among the single drugs in a day (Min-ISD), which is to ensure that the single drug can be taken more reasonably and safely in a day. This prevents poisoning due to the excessive frequency of certain drugs with narrow therapeutic windows and allows the drug administered on time to achieve steady-state blood concentration. To achieve the best efficacy of the drugs, we suggested taking the drugs at the correct time. One of the major issues discussed in this study is how to determine the minimum interval of administration of individual drugs during the day. We determined this parameter by the pharmacokinetic characteristics of the drug such as the peak time of the drug. If a drug has a narrow therapeutic window and the time between the doses is less than the peak time of the drug, the effect of the first peak will be greater than the effect of the time between doses exceeding the peak time of the drug. Moreover, other drugs can affect absorption because the stomach empties in 2 h and the small intestine in 6 h. The indications or dosage of the instructions of some drugs specify a certain time interval before taking them again, such as oxycodone hydrochloride extended-release tablets every 12 h. Moreover, for a drug to be given evenly throughout the day, the 24 h divided by the number of times the drug is taken in a day is defined as the maximum interval between a single drug in a day (Max-ISD).

Combining the abovementioned factors, i.e., the number of times the drug is taken in a day, the meal constraints, Min-ISD, Max-ISD, and chronopharmacology, the recommended medication time was generated for each pharmaceutical product. As shown in Fig. 1, the recommended medication time is the specific time that will be used to systematically rank the medication times. In this study, MTCOD data was eventually established for each pharmaceutical product.

### Data of drug interaction

Drug-drug interactions (DDIs) can lead to changes in drug pharmacokinetics (PK), pharmacodynamics (PD), of which the U.S. Food and Drug Administration (FDA) and the European Medicines Agency (EMA) agencies have issued DDI guidelines for changes in PK. More Data on DDI in the database is available in Supplementary material S1.

### Data of medication time constraint with multi-drug (MTCMD)

DDIs are one of the greatest concerns in rational clinical medication use [18]. In particular, elderly patients with multiple chronic diseases who often take multi-dose medications have a high incidence of potentially inappropriate medication. Approximately 15% of elderly patients have potentially significant DDIs [19, 20]. The establishment of a drug interaction database wherein ingredients are associated with pharmaceutical products can alert physicians to the presence of DDIs in prescribing in time to avoid serious DDIs and adverse drug events.

However, when multiple drugs are prescribed and the risks of using multiple drugs with DDIs outweigh the benefits, we encounter another key question of the study: What is the minimum interval between the two drugs with DDIs to minimize the harmful effect? Therefore, it is important to calculate a minimum interval between two drugs in a day (Min-ITD). The final minimum interval varies depending on the order in which the two drugs are administered. The PK/PD model studies have reported that if aspirin is taken first, taking ibuprofen before 2 h can reduce the antiplatelet effects of aspirin. However, taking ibuprofen after 2 h of taking aspirin does not have any effect. Moreover, taking ibuprofen first and then aspirin after a 24 h interval can affect its antiplatelet effect [21]. Some studies have used a physiologically based pharmacokinetic (PBPK) model to simulate the pharmacokinetic parameters changes of CYP3A4 substrates induced by rifampicin. The results showed that homeostasis is achieved after 2 h of rifampicin administration, the induction effect of rifampicin administration on its substrate was most distinct [22]. At the same time, by calculating the fold change of the pharmacokinetic parameters of the victim drug in the absence and presence of the harmful drug, the range of the parameters that can play an important role was estimated, and then the main process of DDIs was determined [23]. Therefore, this study determined the Min-ITD by establishing PK/PD or PBPK models and calculating the time of the minimum fold change of pharmacokinetic parameters during different DDIs of the two drugs.

If the interaction occurs between the ingredients of two pharmaceutical products, the number of drugs taken in a day, the Min-ITD calculated based on the established PK/PD or PBPK model, and the maximum interval between two drugs in a day (Max-ITD), and, an MTCMD was established for every two pharmaceutical products with DDIs. Examples of this data within the database are presented in Table 1. Within this table, ‘A’ and ‘B’ represent two distinct pharmaceutical products. It’s important to note that variations in the order of administration result in distinct calculations for Min-ITD and Max-ITD.

**Table 1:** Data of medication time constraint with multi-drug with an example

Drugs taken first	Drugs to be taken later	Minimum interval between two drugs in a day (h)	Maximum interval between two drugs in a day (h)
A	B	Length of time	Length of time
B	A	Length of time	Length of time

A, B represent two different pharmaceutical products

If the interaction occurs between the ingredients of the two pharmaceutical products, the number of times the drug is taken in a day, the minimum interval between two drugs in a day calculated from the established PK/PD or PBPK model, and the maximum interval between the two pharmaceutical products that can be administered in a day (this was defined as Max-ITD) were combined. Finally, an MTCMD was established for each of the two pharmaceutical products with DDIs, as shown in Fig. 1.

Information on how the cloud database is implemented and how the data is reviewed can be found in Supplementary material S1. In addition, the functional concepts and implementation of the medication reminder system can also be found in Supplementary material S1.

## Results

### Cloud database

We sorted out instructions and information on 153,916 pharmaceutical products. Among them, 150,376 were domestic, and 96.21% (144,690/150,376) accounted for non-over-the-counter western pharmaceutical products. A total of 3,540 products were imported as shown in Table 2.

**Table 2:** Data content of the medication reminder system

Contents		Counts
Number of pharmaceutical products		153,916
non-OTC western pharmaceutical products		148,230
OTC western pharmaceutical products		1117
non-OTC chinese patent pharmaceutical products		610
OTC chinese patent pharmaceutical products		3959
Medication time constraint with one drug		153,916
Medication time constraint with multi-drug		8,552,712
Drug-drug interaction		
number of pharmaceutical products-ingredient pairs		153,390
number of ingredients		10,615
number of ingredient-ingredient pairs (after removing duplication)		255,708
A source	number of pharmaceutical products-ingredient pairs	489
	number of ingredients	1962
	number of ingredient-ingredient pairs	130,590
	number of foods	18
	number of ingredient-food pairs	463
	number of diseases	519
	number of ingredient-disease pairs	3205
B source	number of pharmaceutical products-ingredient pairs	152,901
	number of ingredients	8653
	number of ingredient-ingredient pairs	366,118

A source, from the drugs.com. B source, from the pharmaceutical product instructions. OTC over-the-counter

MTCOD data on a total of 153,916 products are summarized in Table 2. To unify algorithms that systematically rank the time when drugs are taken, the meal constraints of related drugs and the representation of chronopharmacology were defined by combining original research and pharmaceutical product instructions as shown in Table 3.

**Table 3:** The symbol definition of medication time constraint with one drug in the medication reminder system

Meal constraints or chronopharmacology	Definition	Symbolic representation
Fasting	Refers to taking the medicine 1 h before a meal or 2 h after a meal	-C1;C2
Medication with meals	Refers to taking medication at mealtimes	C0
Take medication before meals	Refers to taking medicine half an hour before meals	-C0.5
	Refers to taking the medicine 3 h before meals	-C3
Take medication after meals	Refers to taking the medicine within 30 min to 1 h after meals	C0.5 ~ C1
	Refers to taking the medicine 3 h after meals	C3
Take before bed	Refers to taking the medicine 15 ~ 30 min before bedtime	-S0.25~-S0.5
Dining time	The system default meal time is 30 min	0.5
Breakfast	The default breakfast meal time of the system is 07:00	ZC0
Lunch	The default lunch time of the system is 12:00	ZHC0
Dinner	The default dinner time of the system is 18:00	WC0
Sleep	The default sleep time of the system is 22:00	S
p.r.n	Refers to the use of medicines in the event of an emergency or other necessary condition	prn

Time unit definition: numbers represent time, the default unit is h, decimals are allowed, accurate to 0.25 h

Letter symbol definition: C means meal, breakfast means ZC, lunch means ZHC, dinner means WC

Operator definition: “-” means negative sign, which means before the time defined by the letter symbol, “+” means after (but the + sign is omitted by default)

Other symbol definitions: “;” means or, “~” means time range

A total of 1,962 ingredients showed 130,590 ingredient-ingredient pairs, and a total of 489 pharmaceutical products were associated with them. As this database contains interactions between drugs, foods, and diseases, we extracted the relevant data, which we will continue to analyze in the future (Table 2).

The database identified 8,653 ingredients, ingredient–ingredient pairs 366,118 items in total, and associated 152,901 pharmaceutical products (Table 2). The hierarchical classification of these drug interactions was as follows: major, moderate, minor, and unknown, according to drugs.com (Table 4).

**Table 4:** Drug interaction classification

Classifications	Definition
Major	Highly clinically significant. Avoid combinations; the risk of the interaction outweighs the benefit
Moderate	Moderately clinically significant. Usually avoid combinations; use it only under special circumstances
Minor	Minimally clinically significant. Minimize risk; assess risk and consider an alternative drug, take steps to circumvent the interaction risk and/or institute a monitoring plan
Unknown	No interaction information available

**Fig. 2:**
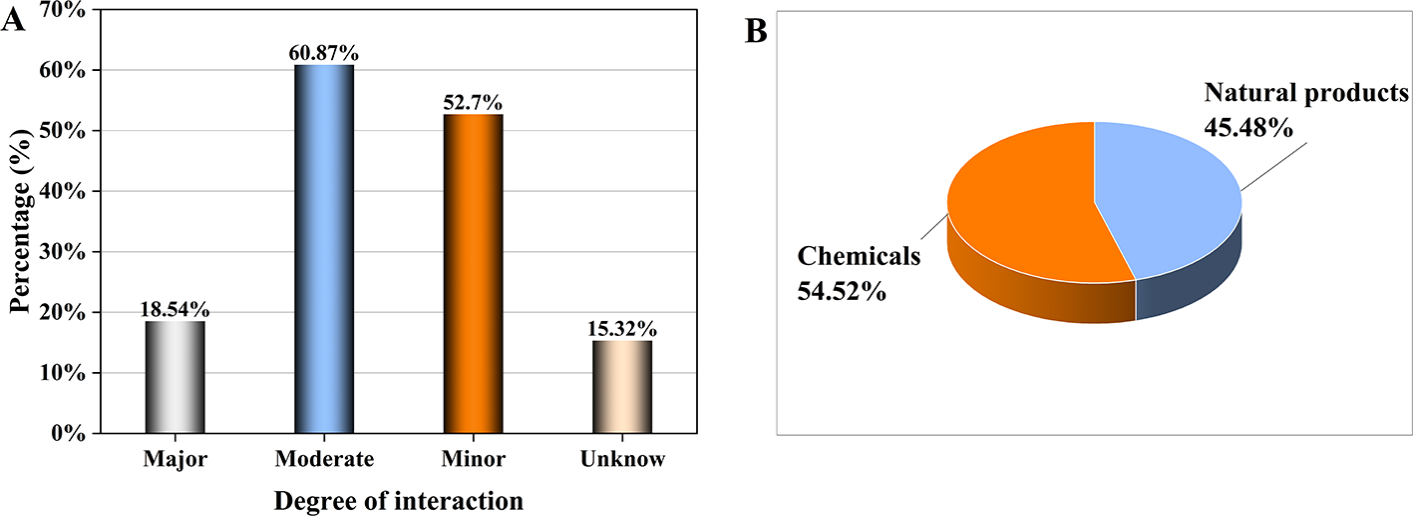
The statistics of drug interaction association based on drug interaction classification (**A**) and ingredients classification (**B**)

The final drug interaction data showed a total of 10,615 ingredients. A total of 255,708 ingredient-ingredient pairs were identified after de-duplicated, which were associated with 153,390 pharmaceutical products (Table 2). The analysis of the ingredient–ingredient pairs showed 60.87% (155,650/255,708) moderate interactions, followed by major interactions, accounting for 18.54% (47,408/255,708) (Fig. 2A). The examination of these ingredients showed that chemicals accounted for 54.51% (5,786/10,615), and Chinese medicine extracts accounted for 43.04% (4,567/10,615). Natural products accounted for 2.44% (269/10,615) as shown in Fig. 2B.

### Web design and implementation

For collecting and sorting data, we set up an open and user-friendly interface of the web database (https://www.hnrongguan.com.cn/yaoxue/login?next=%2Ft_medicine_basic_info). More information about web databases is available in Supplementary material S2.

### Implementation of individualized medication guidance

Based on the constructed web database and concept, we established a medication reminder system and performed a test. The system assigns personalized dosing instructions for the medications taken by each patient. Detailed information can be found in Supplementary material S2.

**Fig. 3:**
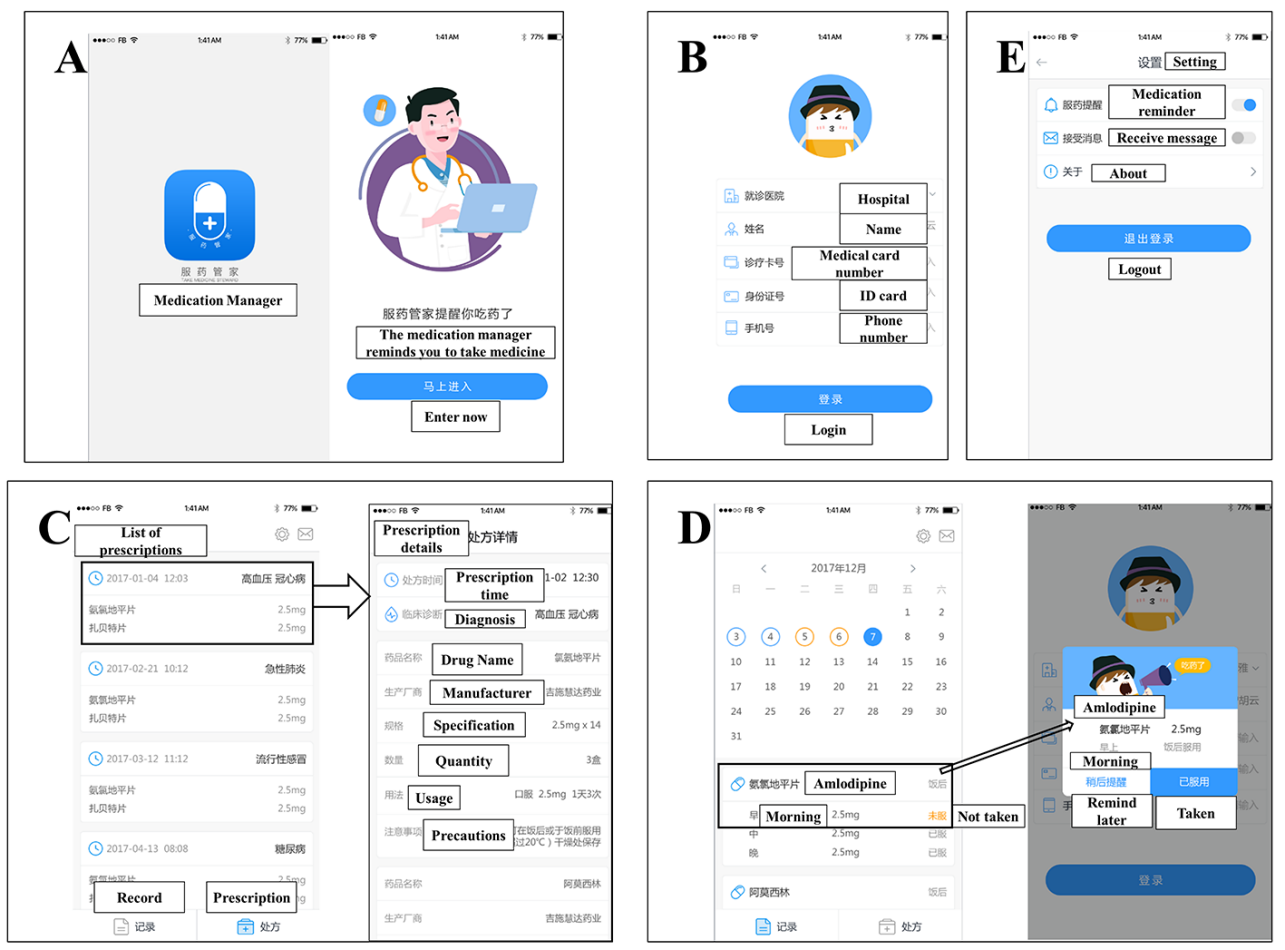
The interface of Medication Manager application (APP): patient use terminal. (**A**) User access. (**B**) User registration. (**C**) Prescription information. (**D**) Reminder for taking medication. (**E**) Settings

We built an APP for patients to use. This APP is called Medication Manager or the patient terminal (Fig. 3A). Through this APP, patients can have real-time feedback with our system to realize a closed-loop of patient management. First, patients need to register and fill in basic personal information including hospital, name, and ID number as shown in Fig. 3B. After registration, patients will see their prescription information and can click on amlodipine besylate tablets to get all information about the pharmaceutical product in the medication guide, including UMS and precautions for the medication (Fig. 3C). The medication time of amlodipine besylate tablets (suggested drug) in the medication guide is correlated with the calendar on the patient’s phone. If the medication has to be taken at 6 am on the same day, the phone will remind the patient via an alarm, and the alarm will stop only after the patient clicks on the Taken option. If a patient is not convenient to take medicine at the suggested time, they can also click on the remind later option, and the phone will remind them 5 min later via an alarm (Fig. 3D). If a patient is not taking the prescribed medication for special reasons, they can turn off the medication-associated reminder and receive a message in the setting option as shown in Fig. 3E.

## Discussion

A study showed that the total coverage of clinical pharmacy services in Chinese primary hospitals is scarce, the utilization rate of clinical pharmacy service software, hardware, and personnel is low, and the quality of pharmacists varies widely [24]. In the face of large and complex pharmaceutical knowledge, medical staff, especially those engaged in clinical pharmaceutical care, should consider how to improve their service level to provide high-quality pharmaceutical care or medication guidance for patients. In the 21st century, artificial intelligence/machine learning can help clinical pharmacists provide more effective medication counseling to patients or other medical personnel, as well as develop clinical pharmacy expert systems tailored to the needs of clinical pharmacy services [25]. Meanwhile, telemedicine and artificial intelligence may reduce repetitive tasks, help assess healthcare professionals whether patients are taking their medications as prescribed, and organize relevant data for a data-driven approach in patient health coaching [26]. Therefore, we built a medication database by summarizing the ontology relationships of pharmaceutical products and developed a medication reminder system with the intelligent generation of UMS as a leading tool.

We have innovatively constructed both MTCOD and MTCMD data by the secondary parsing of the relevant data. When one or two pharmaceutical products can produce adverse effects when administered simultaneously, the interval between their administration should be considered to avoid or reduce their adverse effects. Hence, relevant prediction models should be constructed by integrating multidisciplinary knowledge such as PK/PD, PBPK, and chronopharmacology, which in turn calculates Min-ISD and Min-ITD. Furthermore, managing adverse drug interactions is important in clinical pharmacy services. Many scholars have predicted risks associated with DDIs [27, 28]. Some clinical organizations have published potentially inappropriate medications that should be avoided in the elderly [29]. In the present study, 0.25 M DDIs are summarized concerning 153,390 pharmaceutical products, and information on the specific mechanism of action is provided to guide clinicians when prescribing.

The UMS generated by the medication reminder system facilitates patients’ understanding of medication schedules and improves their medication adherence. It also reduces the work pressure on clinicians and provides a new model of pharmacy services for clinical pharmacists. Additionally, the system improves the efficacy of combination therapy for complex diseases and reduces the occurrence of drug resistance. It has been shown that Zhao et al. developed a novel graph representation learning model, FuHLDR, which first learns low-order representations of drugs and diseases using a graph convolutional network (GNN) and then integrates higher-order connectivity patterns through a meta-path-based strategy. By fusing these representations, FuHLDR employs a network of random vector function links to identify new drug-disease associations. Experimental results show that FuHLDR outperforms existing models, demonstrating its effectiveness in drug repositioning and proving higher accuracy in identifying new drug-disease associations [30]. In addition, Zhao et al. address the over-smoothing problem in existing GNN-based methods to predict drug-target interactions (DTIs) through an improved graph representation learning method, iGRLDTI. iGRLDTI employs a gradient boosting decision-tree classifier to predict new DTIs and outperforms current state-of-the-art methods on a benchmark dataset [31]. In the future, we will use the machine learning approach to analyze the collected patient prescriptions and to find drug combinations with synergistic effects. For drug combinations with potentiation, feature vectors described by public drug databases are concatenated to obtain a certain feature vector for each drug and then fed into classical machine learning or deep learning models for training. These models can be applied to external test sets to assess generalizability or to predict new drug associations. Finally, the predictions are validated by biological experiments or literature searches to explore the relevant biological mechanisms [32].

Patients can access the UMS generated by the system using the APP for accurate medication reminders. Simultaneously, developments in artificial intelligence are updating and improving wearable devices. Currently, the existing wearable sensors are used for fitness and clinical treatment and can help patients at home in the non-invasive monitoring of physiological parameters and vital signs. In the future, the medication reminder system will be associated with wearable devices and apps, which will help timely detect signs of disease deterioration and will be useful in chronic disease management, home care, and other fields of healthcare. In a study, researchers placed wearable sensors on the neck of patients to collect physical data generated by the action of drug ingestion for deep learning to determine whether patients ingested drugs [9].

This study had several limitations. First of all, this system is for patients who are able to use their smartphones normally. Secondly, the accuracy of the data needs to be extensively used clinically, validated and iterated several times. Finally, updates to the application may result in missing or inaccurate patient data.

## Conclusions

To summarize, the medication reminder system intelligently generates clear UMS and sends medication reminders, automatically identifies drug interactions and monitors adverse reactions, and provides in-hospital clinical pharmacy services and bedside pharmacy monitoring as well as out-of-hospital remote monitoring.

### Electronic supplementary material

Below is the link to the electronic supplementary material.

Supplementary Material 1

Supplementary Material 2

Supplementary Material 3

## Data Availability

The datasets used and/or analyzed during the current study are available from the corresponding author on a reasonable request.

## Code Availability

The code that supports the findings of this study are available from the corresponding author upon reasonable request.

## Declarations

### Moral Declaration

The paper was not considered by another journal, and the results here have not been published before. All authors met the criteria for authorship and agreed to publish.

## Abbreviations

IOM: Institute of Medicine
UMS: Universal medication schedule
MTCOD: Medication time constraint with one drug
Min-ISD: Minimum interval between single drug in a day
Max-ISD: Maximum interval between single drug in a day
DDIs: Drug-drug interactions
PK: Pharmacokinetics
PD: Pharmacodynamics
FDA: Food and Drug Administration
EMA: European Medicines Agency
ADME: Absorption: distribution: metabolism: and excretion
MTCMD: Medication time constraint with multi-drug
Min-ITD: Minimum interval between two drugs in a day
Max-ITD: Maximum interval between two drugs in a day
PBPK: Physiologically based pharmacokinetic
ID: Identification
GNN: Graph convolutional network
DTIs: Drug-target interactions

## References

[1] Al Bawab AQ, Al-Qerem W, Abusara O, Alkhatib N, Mansour M, Horne R. What are the Factors Associated with Nonadherence to medications in patients with chronic diseases? Healthc (Basel). 2021;9(9). 10.3390/healthcare9091237 PMC8469667 34575011

[2] Choi EPH. A pilot study to evaluate the acceptability of using a Smart Pillbox to Enhance Medication Adherence among Primary Care patients. Int J Environ Res Public Health. 2019;16(20). 10.3390/ijerph16203964 PMC6843901 31627440

[3] Marquez Contreras E, Marquez Rivero S, Rodriguez Garcia E, Lopez-Garcia-Ramos L, Carlos Pastoriza Vilas J, Baldonedo Suarez A (2019). Specific hypertension smartphone application to improve medication adherence in hypertension: a cluster-randomized trial. Curr Med Res Opin.

[4] Brown MT, Bussell J, Dutta S, Davis K, Strong S, Mathew S (2016). Medication adherence: Truth and consequences. Am J Med Sci.

[5] Ho PM, Bryson CL, Rumsfeld JS (2009). Medication adherence: its importance in cardiovascular outcomes. Circulation.

[6] Wolf MS, Davis TC, Curtis LM, Bailey SC, Knox JP, Bergeron A (2016). A patient-centered prescription drug label to promote appropriate medication use and adherence. J Gen Intern Med.

[7] McManus E, McCarthy S, Carson R, Sahm LJ (2018). Impact of a Universal Medication schedule on rationalising and understanding of medication; a randomised controlled trial. Res Social Adm Pharm.

[8] Bailey SC, Wolf MS, Lopez A, Russell A, Chen AH, Schillinger D (2014). Expanding the Universal Medication schedule: a patient-centred approach. BMJ Open.

[9] Mason M, Cho Y, Rayo J, Gong Y, Harris M, Jiang Y (2022). Technologies for Medication Adherence Monitoring and Technology Assessment Criteria: Narrative Review. JMIR Mhealth Uhealth.

[10] Morawski K, Ghazinouri R, Krumme A, Lauffenburger JC, Lu Z, Durfee E (2018). Association of a Smartphone Application with Medication adherence and blood pressure control: the MedISAFE-BP Randomized Clinical Trial. JAMA Intern Med.

[11] Mira JJ, Navarro I, Botella F, Borras F, Nuno-Solinis R, Orozco D (2014). A Spanish pillbox app for elderly patients taking multiple medications: randomized controlled trial. J Med Internet Res.

[12] McManus RJ, Little P, Stuart B, Morton K, Raftery J, Kelly J (2021). Home and online management and evaluation of blood pressure (HOME BP) using a digital intervention in poorly controlled hypertension: randomised controlled trial. BMJ.

[13] Browne SH, Umlauf A, Tucker AJ, Low J, Moser K, Gonzalez Garcia J (2019). Wirelessly observed therapy compared to directly observed therapy to confirm and support tuberculosis treatment adherence: a randomized controlled trial. PLoS Med.

[14] Hafezi H, Robertson TL, Moon GD, Au-Yeung KY, Zdeblick MJ, Savage GM (2015). An ingestible sensor for measuring medication adherence. IEEE Trans Biomed Eng.

[15] Wachira C, Osebe S, Ogallo W, Walcott-Bryant A (2019). Enhancing Guideline-based prescribing and personalized medication scheduling. Stud Health Technol Inf.

[16] Bailey SC, Persell SD, Jacobson KL, Parker RM, Wolf MS (2009). Comparison of handwritten and electronically generated prescription drug instructions. Ann Pharmacother.

[17] Lemmer B (2005). Chronopharmacology and controlled drug release. Expert Opin Drug Deliv.

[18] Wienkers LC, Heath TG (2005). Predicting in vivo drug interactions from in vitro drug discovery data. Nat Rev Drug Discov.

[19] Huang Y, Zhang L, Huang X, Liu K, Yu Y, Xiao J (2020). Potentially inappropriate medications in Chinese community-dwelling older adults. Int J Clin Pharm.

[20] Qato DM, Wilder J, Schumm LP, Gillet V, Alexander GC (2016). Changes in prescription and Over-the-counter Medication and Dietary supplement use among older adults in the United States, 2005 vs 2011. JAMA Intern Med.

[21] Awa K, Satoh H, Hori S, Sawada Y (2012). Prediction of time-dependent interaction of aspirin with ibuprofen using a pharmacokinetic/pharmacodynamic model. J Clin Pharm Ther.

[22] Baneyx G, Parrott N, Meille C, Iliadis A, Lave T (2014). Physiologically based pharmacokinetic modeling of CYP3A4 induction by rifampicin in human: influence of time between substrate and inducer administration. Eur J Pharm Sci.

[23] Duan JZ (2010). Drug-drug interaction pattern recognition. Drugs R D.

[24] Yao D, Xi X, Huang Y, Hu H, Hu Y, Wang Y (2017). A national survey of clinical pharmacy services in county hospitals in China. PLoS ONE.

[25] Corrigan BW (2020). Artificial Intelligence and Machine Learning: Will Clinical Pharmacologists be needed in the Next Decade? The John Henry Question. Clin Pharmacol Ther.

[26] Eggerth A, Hayn D, Schreier G (2020). Medication management needs information and communications technology-based approaches, including telehealth and artificial intelligence. Br J Clin Pharmacol.

[27] Nyamabo AK, Yu H, Shi JY. SSI-DDI: substructure-substructure interactions for drug-drug interaction prediction. Brief Bioinform. 2021;22(6). 10.1093/bib/bbab133 33951725

[28] Zheng Y, Peng H, Zhang X, Zhao Z, Gao X, Li J (2019). DDI-PULearn: a positive-unlabeled learning method for large-scale prediction of drug-drug interactions. BMC Bioinformatics.

[29] By the American Geriatrics Society Beers Criteria Update Expert P. American Geriatrics Society (2019). 2019 updated AGS Beers Criteria(R) for potentially inappropriate medication use in older adults. J Am Geriatr Soc.

[30] Zhao BW, Wang L, Hu PW, Wong L, Su XR, Wang BQ (2024). Fusing higher and lower-Order Biological Information for Drug Repositioning via graph representation learning. Ieee T Emerg Top Com.

[31] Zhao BW, Su XR, Hu PW, Huang YA, You ZH, Hu L. iGRLDTI: an improved graph representation learning method for predicting drug-target interactions over heterogeneous biological information network. Bioinformatics. 2023;39(8). 10.1093/bioinformatics/btad451 PMC10397422 37505483

[32] Wu L, Wen Y, Leng D, Zhang Q, Dai C, Wang Z et al. Machine learning methods, databases and tools for drug combination prediction. Brief Bioinform. 2022;23(1). 10.1093/bib/bbab355 PMC8769702 34477201

