# Supplementary material for: Construction and application of medication reminder system: intelligent generation of universal medication schedule
Source: BioData Min. 2024 Jul 15;17:23. doi: 10.1186/s13040-024-00376-y (PMC11247871; doi:10.1186/s13040-024-00376-y)
Supplement: Supplementary file 1 — Supplementary Material 1 [file 13040_2024_376_MOESM1_ESM.docx]

**Basic drug information**

The dosage section was categorized according to the use of the general population of the drug, special populations, and different diseases. Each category was then subdivided into usage, starting dose, average dose, and maximum dose.

The pharmacokinetics section was classified based on the absorption, distribution, metabolism, and excretion of the drug. Absorption included the site of absorption, the effect of food on absorption, the onset of action, maintenance of action, time to peak plasma concentration, and bioavailability. The distribution included the site of distribution, volume of distribution, and plasma protein binding rate. Metabolism included metabolic site, metabolic enzymes, metabolites, steady-state plasma drug concentration, and effective plasma drug concentration. Excretion included site, rate, and half-life of elimination.

Adverse reactions were classified as general adverse reactions and serious adverse reactions, which included adverse reactions to the digestive system, nervous system, respiratory system, cardiovascular system, hematological system, digestive system, and skin.

The remaining content of pharmaceutical product instructions was streamlined by professionals. The content from the original studies was searched in PubMed based on the ingredient of the pharmaceutical products to determine which studies reported drug-dose correlation and which studies were appropriate for the education on medication and disease for the patient. The search formula is the ingredient of each pharmaceutical product.

**Data of drug interaction**

Drug-drug interactions (DDIs) can lead to changes in drug pharmacokinetics (PK), pharmacodynamics (PD), of which the U.S. Food and Drug Administration (FDA) and the European Medicines Agency (EMA) agencies have issued DDI guidelines for changes in PK. Typically, PK is characterized by changes in the drug-time profile, which can be mediated by changes in the absorption, distribution, metabolism, and excretion (ADME) processes of one drug (victim) by another compound (perpetrator) when administered simultaneously ^1^. DDIs can trigger chronic clinical effects by increasing toxicity and reducing therapeutic efficacy. Therefore, physicians should avoid harmful drug combinations while prescribing to reduce the pharmacogenic damage caused by DDIs ^2^.

Drug interaction data was extracted and structured based on the open drug interaction database drugs.com. It provided much information such as mechanisms of interaction, classes, recommendations, and references for medical professionals.

Information on DDIs was abundant in pharmaceutical product instructions. No uniform structure was available for this section. Some were the interactions of pharmaceutical products with a specific ingredient, some contents of this section were not uniformly structured, and some were the interactions of ingredients with similar pharmacological effects. The precautions and contraindications of pharmaceutical products were also in the drug interaction section. Therefore, this section was rich but disorganized, and we structured it based on the basic description of ingredient-ingredient, interaction mechanism, interaction class, treatment recommendation, and ingredient description. In addition, information was consulted for controversial interactions, and relevant studies with literature rank classification and descriptions were included.

**Online database implementation**

The Python Web Framework Flask and Elasticsearch real-time search engine were used. The Web interface was developed using HTML5, JavaScript, jQuery, and Layui. All data was stored and managed using the MySQL database and Redis Cache database.

**Advisory panel**

The information extracted from pharmaceutical product instructions and PubMed was thoroughly reviewed by registered physicians and pharmacists at the Xiangya Hospital of Central South University. The review process is illustrated in **Figure S1**. In order to make the data more accurate and reasonable, raw data were first reviewed by two review teams. If their opinion reached a consensus, the data was finally determined. Otherwise, a third review team intervened and the three review teams jointly confirmed until an opinion was reached with consensus. Each review team was composed of 2 physicians and 2 pharmacists, and their designations were deputy directors or above. The engineers from the National Supercomputing Center in Changsha provided professional technical support to ensure the smooth development of the system.

**The construction of medication reminder system**

*Functional concept*

The system is used in hospital outpatient clinics or other healthcare facilities, especially in middle and lower developing countries. When a patient receives a prescription from a doctor, the most difficult and important thing to understand is the dosage of the medicine. Instead of stating how many tablets are to be taken at a time, prescriptions are often written as, for example, 5 mg and 0.1 g. There are also no precautions regarding the timing of the medication, food interactions, drug interactions, and so on. This is difficult to understand and error prone for older, chronically ill and less educated patients. The time of day to take the medicine is also a concern. Based on these problems, we aimed to construct a system to automatically schedule the time and dosage of drugs, and finally generated the UMS.

The scheduling of drugs requires a basic time frame, which included the minimum time unit for the medication (hour, which can be further refined according to actual needs such as half an hour), typical meal times (default 7, 12, and 18 h), and rest periods (22 to 6 the next day). The basic time frame is shown in **Figure S2A**, which shows the following: 1) Gray represents the rest time. If it is not necessary, medication will not be arranged during this time. 2) Orange indicates meal time. If the medication should be taken with meals, it should be arranged at this time. If the medication should be taken before or after meals, it should be taken according to the time interval with this time. 3) Blue indicates working hours, and the medication time should be taken at this time. Different patients often have different sleep schedules (different occupations and different age groups have different meal and sleep times). To achieve customized precision medicine, the above time frame should be adjusted by consulting the patients.

After determining the time frame, drugs were selected from the established drug database according to the patient’s prescription and were added to the prescription list. After the secondary analysis of the MTCOD data, the drug time was decided. As shown in **Figure S2B**, the red color represents the drug taking time corresponding to Drug A or B. Drug interactions were not checked when the prescription list contained a single drug. Drug interactions were checked if the list contained more than two drugs. We assumed that Drug B did not interact with Drug A.

Then Drug C was added, and the system automatically checked for interactions among Drug C, Drug A, and Drug B, which were already in the prescription. In this case, we assumed that C did not interact with A but interacted with B. Hence, the medication time for B or C must be adjusted as shown in **Figure S2C**. At this point, the system used the MTCMD data to determine the interval between Drug B and Drug C administration.

After all medications have been added and programmed, the dosing times for all medications are reviewed by the medical staff for compliance with MTCOD and MTCMD. Moreover, system will show a pop-up warning regarding drug interactions, and users can see information such as the specific mechanism of drug action. Hence, based on the overall drug information, medical staff can decide on drug taking time according to patient conditions and their own needs for fine adjustment. Finally, the prescription was exported for a subsequent medication reminder as shown in **Figure S2D**.

**The medication reminder system implementation**

The system was built based on the Python web framework of flask 1.1.0. The web interface was developed using Vue 3.0 and Element 2.15.0. All data were stored and managed using MySQL 5.7. The website was tested thoroughly to ensure its functionality across multiple operating systems and web browsers.


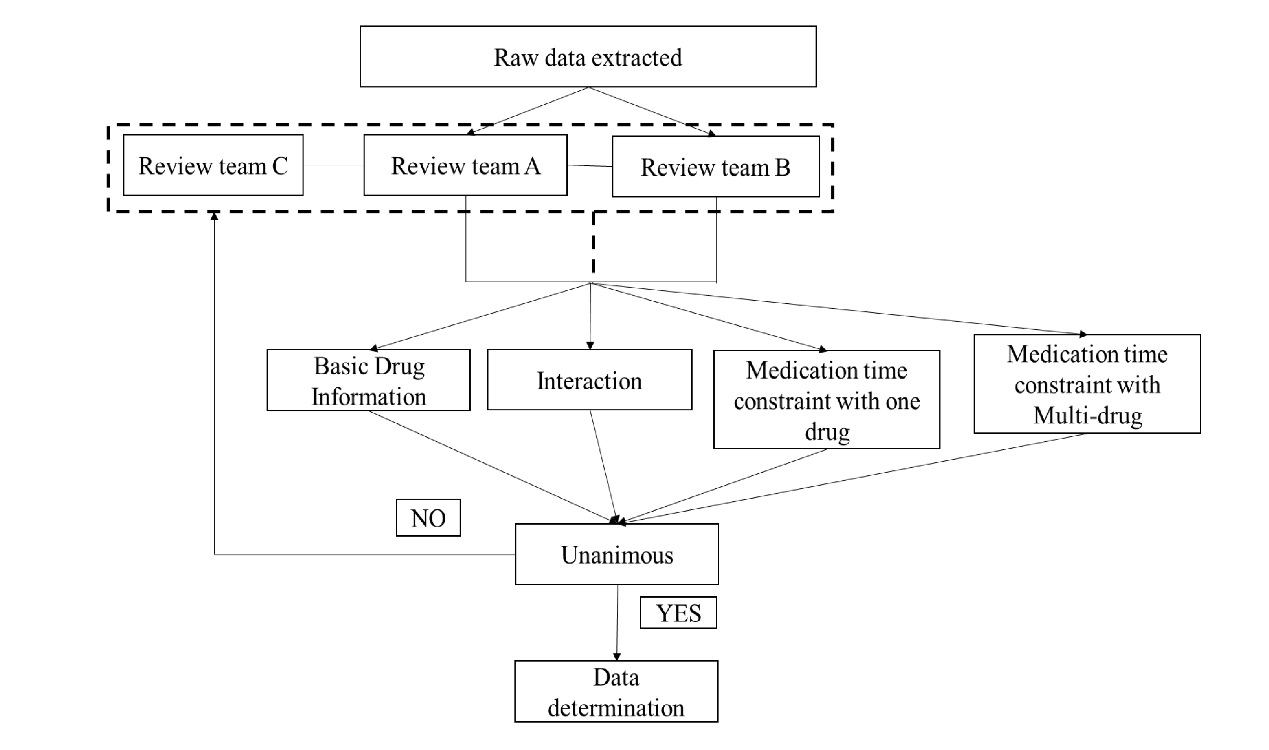
**Figure** **S1** Data review process of the medication reminder system.


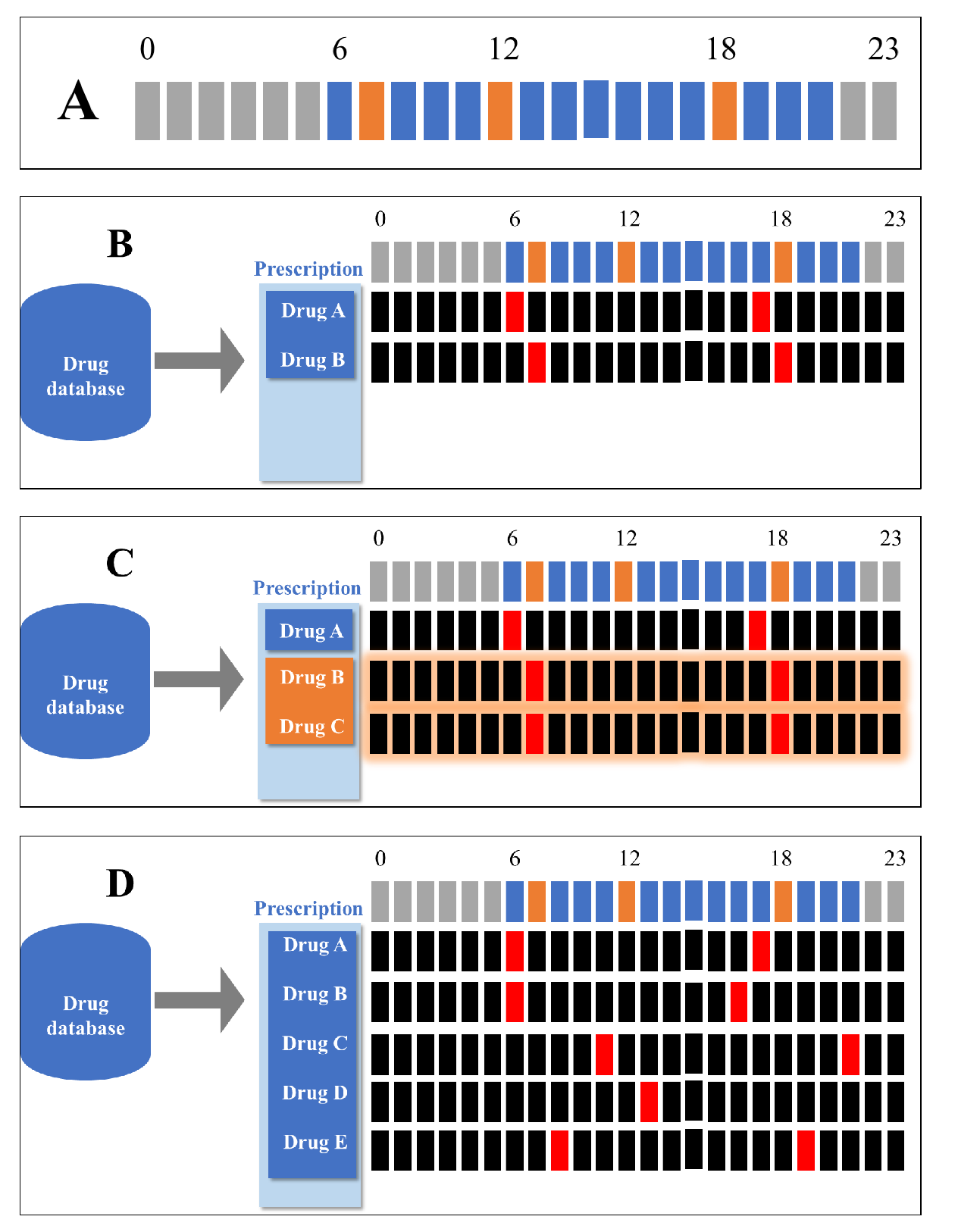
**Figure S2** The basic idea of scheduling the medication time. **(A)** The basic time frame of the average person in a day. Gray denotes rest time, blue denotes working time, and orange denotes meal time. **(B)** According to the prescription prescribed by the doctor, the medication time of the corresponding drugs in the drug database was searched and arranged. **(C)** At the same time, whether there is an interaction between different drugs are searched, such as Drug A and Drug B. **(D)** Finally, the timing of the prescription was confirmed by the pharmacist or medical staff.

**References:**

1. Prueksaritanont T, Chu X, Gibson C, et al. Drug-drug interaction studies: regulatory guidance and an industry perspective. AAPS J 2013;15(3):629-45, doi:10.1208/s12248-013-9470-x

2. Qiu Y, Zhang Y, Deng Y, et al. A Comprehensive Review of Computational Methods for Drug-drug Interaction Detection. IEEE/ACM Trans Comput Biol Bioinform 2021;PP(doi:10.1109/TCBB.2021.3081268
