# Supplementary material for: Construction and application of medication reminder system: intelligent generation of universal medication schedule
Source: BioData Min. 2024 Jul 15;17:23. doi: 10.1186/s13040-024-00376-y (PMC11247871; doi:10.1186/s13040-024-00376-y)
Supplement: Supplementary file 2 — Supplementary Material 2 [file 13040_2024_376_MOESM2_ESM.docx]

**Web design and implementation**

Users could apply for an account and password from the developer by mentioning their reasonable requirements and could check it after logging in (**Figure S1A**).

The content of instructions in each field was processed structurally to obtain basic drug information. Users can retrieve pharmaceutical product names, manufacturers, specifications, and ingredients, individually or in combination, to get a specific pharmaceutical product or all pharmaceutical products that contain a certain ingredient. Each pharmaceutical product in the database has an identification (ID) number for better identification, and each row represents a pharmaceutical product. Drag the lower bar to view all the remaining fields, and double-click any row to view all the fields of a pharmaceutical product corresponding to that row (**Figure S1B**).

We generated MTCOD data for each pharmaceutical product. The data included the meal constraint of a pharmaceutical product, Min-ISD and Max-ISD, recommended medication time period, and chronopharmacology. Users can retrieve the name or number of doses taken in a day individually and can get all data of a pharmaceutical product or all pharmaceutical products for a specific number of doses. In the case of combined retrieval, the data of the time of taking a pharmaceutical product for a certain drug can be obtained as shown in **Figure S1C**.

For two pharmaceutical products whose ingredients interact with each other, individually or in combination, enter the number of ingredients used first, the number of ingredients used first, the number of ingredients used second, and the number of ingredients used second to obtain Min-ITD and Max-ITD of the two pharmaceutical products (**Figure S1D**).

Each pharmaceutical product was associated with the contained ingredient or multiple associations in the case of a compound ingredient, indicating multiple lines of data. A user can enter the drug name and/or ingredient in Chinese and English (**Figure S1E**) and the drug name and/or ingredient in Chinese (**Figure S1I**) for retrieval. They can also retrieve ingredient–ingredient interactions by entering single or paired Chinese and English ingredients and/or interaction levels (**Figure S1F**) and single or paired Chinese ingredients (**Figure S1J**) to access drug interaction information. In addition to drug interactions, a user can enter ingredients in Chinese and English, disease names and/or interactions in Chinese and English (**Figure S1G**), and ingredients and/or food names and/or interactions in Chinese and English (**Figure S1H**) in case of any query about information regarding ingredient–disease and ingredient–food interactions.

**Implementation of individualized medication guidance**

The hardware equipment includes a computer, optical character recognition (OCR) scanner, and printer as shown in **Figure S2B**. Consider a situation where after a patient’s outpatient visit, the doctor writes a prescription containing four pharmaceutical products: digoxin tablets, acarbose tablets, sucralfate suspensions, and esomeprazole-enteric-coated tablets. After receiving the paper prescription, the patient can visit the medication reminder system (**Figure S2A**).

After the prescription information is input, the system will review the prescription (**Figure S2C**). In addition, if the patient does not have a paper prescription, the patient’s stated current medication can also be manually entered at this stage. Due to certain deviations in the identification of picture information, the patient needs to manually click the Automatic Matching button. In addition to checking the basic information of patients, checking the number of times and dosage of drugs taken each time is most important. In the above example, the medical staff confirmed the dosage and time as follows- digoxin tablets: once a day, half a tablet at a time; acarbose tablets: three times a day, one tablet at a time; sucralfate suspension: three times a day, 10 mL at a time; and esomeprazole-enteric-coated tablets: once a day, two tablets at a time. Then, click on the submit option.

Then the dosing time was adjusted (**Figure S2D**). On this interface, medical staff can first ask patients about their meal and rest times and then compare them with the basic time frame formulated by us. For example, the breakfast time is 7 o ’clock, and a patient’s self-reported time is 9 o ’clock. If the two are inconsistent, they can choose to adjust the time to 9 o ’clock or follow the basic time frame formulated after consultation with the patient. After the time frame is determined, check the medication timetable arranged by the system; references and comparisons are based on the data of time frame, MTCOD, and MTCMD; simultaneously, if the current arranged medication time cannot meet these data, the patient will be reminded in the Medication Restriction Reminder box. In addition, if medical personnel want to specifically view the pharmaceutical product information, they can double-click the pharmaceutical product (digoxin tablet in the example here) in the first row in the Timetable box and the first row in the drug interaction box. The basic drug information of digoxin tablets and the specific information on the interaction between digoxin tablets and acarbose tablets can be viewed, respectively. The patient can also click on Prescription Comparison to see how patients of the same class used to adjust their medication times when they had the same or a similar prescription. After confirming all the information, digoxin tablets and esomeprazole-enteric-coated tablet doses were prescribed to take at 6 o ’clock, acarbose tablet doses were prescribed to take at 7 o ’clock, 12 o ’clock, and 18 o ’clock, and sucralfate suspension doses were prescribed to take at 11 o ’clock, 17 o ’clock, and 22 o ’clock (**Figure S2E**).

In addition, after you click on the prescription comparison button, the system will retrieve previously adjusted prescriptions that are similar to the current prescription. By comparing or referring to previous prescriptions, medical staff can also adjust the time of current prescription drugs more quickly or accurately (**Figure S2E**).

Furthermore, consider the edit medication guide, which is user-friendly and easy for the patient to view. The basic content is automatically put into the patient education box, and medical staff can add or delete personalized information according to the patient’s clinical diagnosis and other personal information. All blank field boxes can also be added or deleted as shown in **Figure S2F**. After editing the medication information, click on the confirm button to generate the final medication guide. The patient will get the document in the PDF that can be renamed (**Figure S2G**).

The medication guide contains two pages. The first page is the patient’s personal information and the attendant trader (**Figure S2H**). The attendant table is equivalent to UMS, indicating that one amlodipine besylate tablet of 5 mg should be taken orally at 6 o ’clock before breakfast. This way, patients will have a clear understanding of the medicine to take, its time, and its dose. The second page is the precautions for medication for the patient. If there is no content in some fields in the edit medication guide stage, it will not appear in the final precautions for medication section. So, in the end only three fields are shown: patient education, precautions, and food interactions. The patient can print a hard copy of the final medication guide and give it to the doctor who wrote the prescription to sign. Patients can also scan the two-dimensional code, get an electronic version of the medication guide, and download an APP.


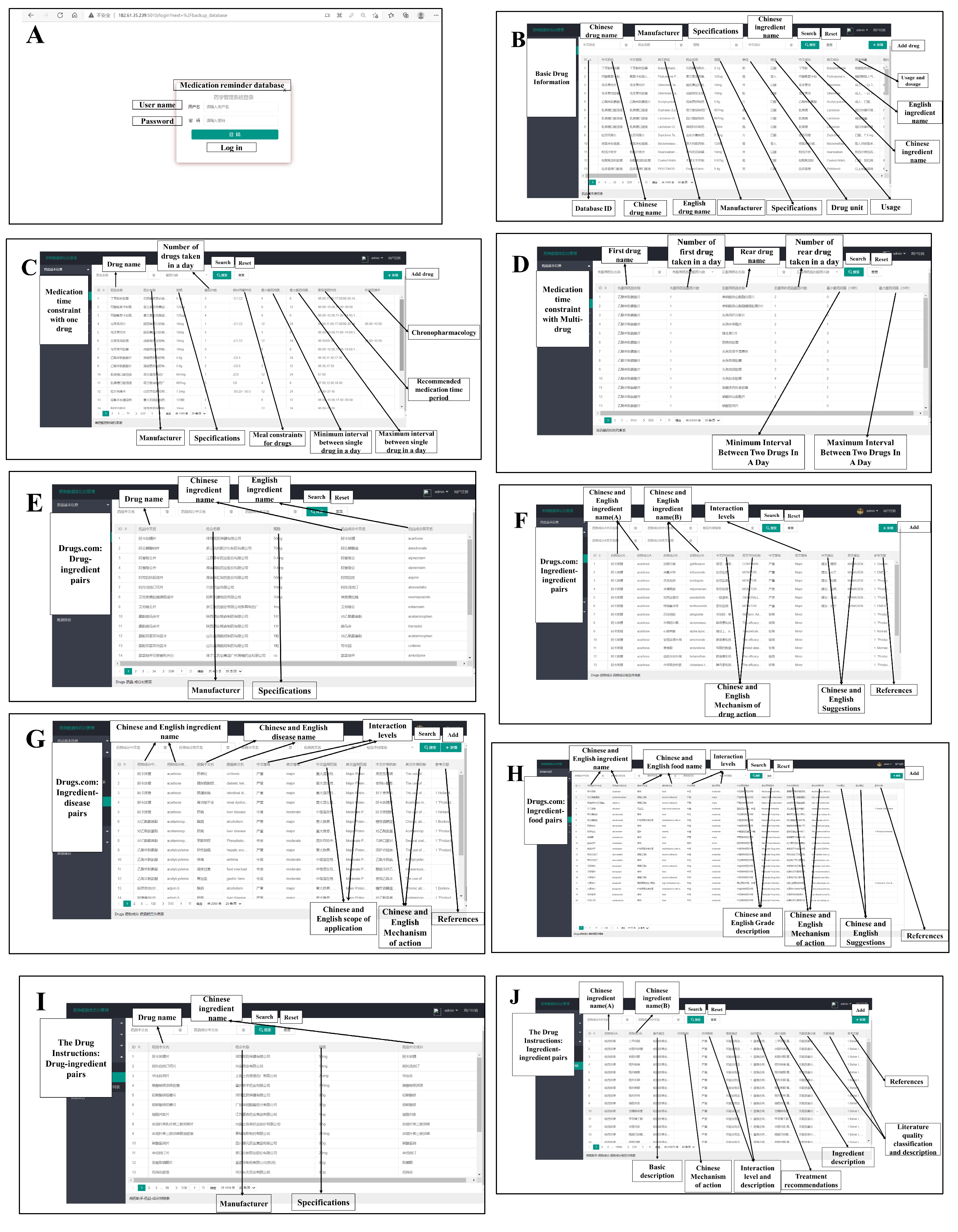


**Figure S1** The web-database interface of medication reminder system. (**A**) The login interface of the database. (**B**) Data of basic drug information. (**C**) Data of medication time constraint with one drug. (**D**) Data of medication time constraint with multi-drug. Rear drug means drugs for after-use. (**E)** Data of pharmaceutical products-ingredient pairs from drugs.com. (**F**) Data of ingredient-ingredient pairs from drugs.com. (**G**) Data of ingredient-disease pairs from drugs.com. (**H**) Data of ingredient-food pairs from drugs.com. (**I**) Data of pharmaceutical products-ingredient pairs from instructions. (**J**) Data of ingredient-ingredient pairs from instructions.


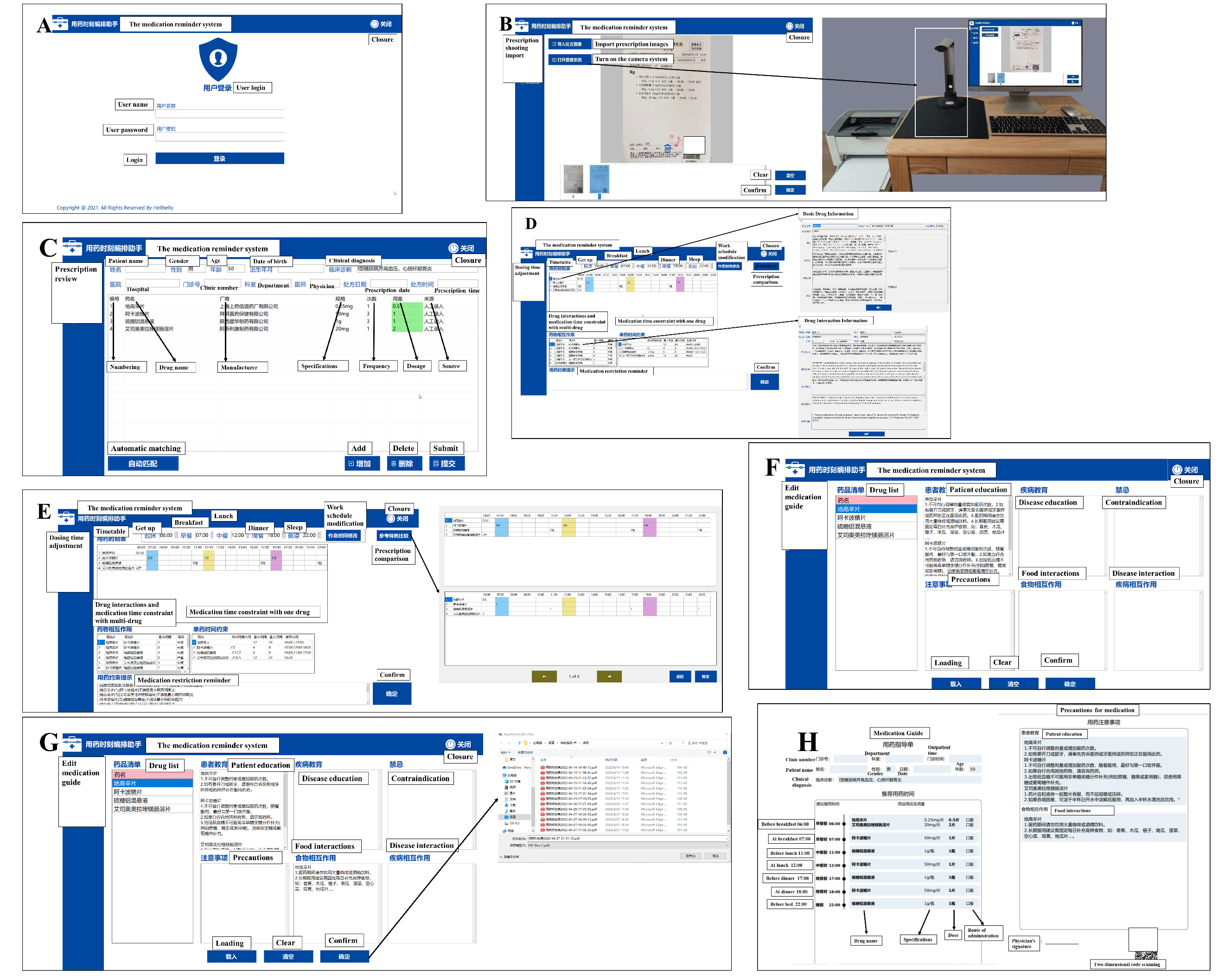


**Figure S2** The medical staff interface of medication reminder system. (**A**) User login. (**B**) Prescription entry. (**C**) Prescription information check. (**D**) The pharmaceutical products inspection and medication adjustment. (**E**) Prescription comparison. (**F**)The medication guide content adjustment. (**G**) The medication guide pdf document generation. (**H**) The medication guides.
